# Electrical Stimulation Activates Fibroblasts through the Elevation of Intracellular Free Ca^2+^: Potential Mechanism of Pelvic Electrical Stimulation Therapy

**DOI:** 10.1155/2019/7387803

**Published:** 2019-04-21

**Authors:** Suting Li, Danhua Lu, Jianming Tang, Jie Min, Ming Hu, Yang Li, Yaodan Liu, Linlin Wang, Cheng Liu, Li Hong

**Affiliations:** Department of Gynecology and Obstetrics, Renmin Hospital of Wuhan University, Wuhan, Hubei, China

## Abstract

Ca^2+^ is an important ion in response to electrical stimulation (ES) and acts as second messenger in the regulation of various physiological processes. Pelvic floor electrical stimulation (PES) is a low-voltage clinical application, available for urinary incontinence (UI) treatment. Fibroblasts, as the main cellular component of vaginal wall and pelvic ligament, play an important role in the maintenance of pelvic health. We studied the effect of ES on fibroblasts in this study. ES was conducted with electrotaxis chambers on L929 fibroblast and the ES parameter was 100 mV/mm×2h. The results showed that ES increased intracellular Ca^2+^ concentration, promoted the expression of PCNA, CyclinB1, and CyclinD1, and increased the proportion of cells in S and G2 phages. After ES, fibroblasts get activated and proliferated. Besides, BAPTA-AM, a membrane permeated chelator for intracellular free Ca^2+^, partially inhibited the effect of ES on fibroblasts activation and proliferation promotion. Furthermore, we elucidated that Ca^2+^, as a second messenger and upstream signal for Smads and Akt signaling, regulated ES-induced nuclear translocation of smad2/3, phosphorylation of smad2/3, Akt, and GSK3*β*. Finally, we validated the effect of ES on PES mouse model. The results indicated that PES promoted the activation and proliferation of fibroblasts* in vivo*. In conclusion, we verify that ES can elevate the concentration of intracellular Ca^2+^ and activate its downstream signaling and then promote the activation of fibroblasts, which may be one of the mechanisms of PES therapy.

## 1. Introduction

Pelvic floor disorders (PFD) are common diseases among middle and aged women [1]. Among these disorders, urinary incontinence (UI) and pelvic organ prolapse (POP) are common types, which affect the quality of life of women throughout the world and cause huge public health burden [2]. The occurrence of PFD results from the weakness of pelvic supporting tissues such as the levator ani muscle, ligaments, and fascia. The anterior vaginal wall is of great importance for the support of urethra and the normal function of urinary continence because urethra and vesical neck are closely attached to it [3]. Slackness of vaginal wall leads to supporting failure of urinary tract, which is one of the most important underlying pathomechanisms of UI.

Fibroblasts are the most significant cellular component of vaginal wall. The abnormal activity of fibroblasts may contribute to the genesis and progress of PFD. Previous studies demonstrated that there are alterations of collagen levels and metalloproteinase activities in uterine ligaments from POP patients [4]. Our previous research suggests that uterosacral ligament fibroblasts from POP patients show compromised collagen synthesis [5]. Effective treatments aim to regulate the activation, proliferation, and ECM synthesis deserving more attention for the therapy.

At present, invasive surgical treatment is the main choice for advanced PFD, along with inevitable side effects. In addition to surgery, physical therapy such as pelvic floor electrical stimulation (PES) and pelvic floor muscle training plays an important role in the prevention and therapy of mild PFD symptoms [6]. According to the guidelines from European Association of Urology in 2017, PES is one of the most available physical therapies for UI [7], and physical therapies should be offered as a first-line treatment to improve women's health outcomes [8]. PES is a low-voltage clinical application, available for UI treatment with high levels of evidence [9]. Many studies have verified that PES can effectively alleviate urinary incontinence symptoms compared with single pelvic floor muscle training [10]. The study of the mechanism of electrical stimulation (ES) therapy is of great significance for effective intervention and prevention of UI and is expected to fundamentally reduce the incidence of this disease.

Electrophysiological study reveals the presence of voltage-sensitive calcium channels in fibroblasts [11]. Levels of free Ca^2+^ rise in fibroblasts under ES [12], thus causing Ca^2+^-sensitive processes such as cytoskeletal organization, cell motility, and cell growth. Our previous research indicates that ES improved the outcome of UI symptoms using stress urinary incontinence (SUI) mouse model [13], and Smads signaling may be involved in this process. Furthermore, Akt signaling is the pathway related with fibroblasts activation, proliferation, and migration [14–16]. To further uncover the therapeutic mechanism of PES, we investigated the effects and underlying mechanisms of ES on collagen synthesis, activation of smad2/3 signaling, and Akt/GSK3*β* signaling,, as well as the involvement of Ca^2+^. in C57/BL6 mice and L929 fibroblasts with or without ES. Our findings suggest that ES can activate fibroblasts via the promotion of intracellular Ca^2+^, and smad2/3 and Akt/GSK3*β* signaling might be involved in this process.

## 2. Materials and Methods

### 2.1. Agents and Antibodies

RPMI 1640 medium and Phosphate Buffer Saline (PBS) were purchased from Genom Biotech Co. Ltd. (Hangzhou, China). Fetal Bovine Serum (FBS) was purchased from Gibco® (Waltham, USA). Antibodies GAPDH (ab181602), COL3A1 (ab7778), *α*-SMA (ab124964), CyclinD1 (ab16663), and CyclinB1 (ab181593) were obtained from Abcam plc. (Cambridge, UK). PCNA (10205-2-AP) and Vimentin (60330-1-lg) were purchased from Proteintech Group (Chicago, IL). Akt (cst2966), GSK3*β* (cst12456s), p-Akt (cst4058), p-GSK3*β* (cst555T), smad2/3 (cst72255), p-smad2 (cst18338), and p-samd3(cst9520) were purchased from Cell Signaling Technology Inc. (Danvers, MA, USA). Goat Anti-Mouse IgG (H+L) Secondary Antibody and FITC Conjugate (BA1101), Goat Anti-Rabbit IgG (H+L) Secondary Antibody and FITC Conjugate (BA1105), and Goat Anti-Rabbit IgG (H+L) Secondary Antibody and Cy3 Conjugate (BA1032) were purchased from Boster Biotech Co. Ltd. (Wuhan, China). Goat Anti-Rabbit and Goat Anti-Mouse Secondary Antibodies (P/N 925 32211 and P/N 925 32210) were purchased from LI-COR Biosciences (Lincoln, NE, USA). BAPTA-AM (B4758) was obtained from Apexbio, USA, and it was prepared as 25 mM stock solutions in dimethyl sulfoxide (DMSO). Fluo-3AM (39294) was purchased from Sigma Aldrich. Cell-Light™ EdU Apollo®488* In Vitro* Imaging Kit (C10310-3) was purchased from RiboBio Inc. (Guangzhou, China). Cell Cycle and Apoptosis Analysis Kit (C1052) was purchased from Beyotime Biotech Co. Ltd. (Suzhou, China). Transwell insert chambers were purchased from Corning Inc. (NY, USA).

### 2.2. Cell Culture and ES Treatment

L929 cells (CX0187) were purchased from Boster Biotech Co. Ltd. (Wuhan, China) and cultured in RPMI 1640 supplemented with 10% FBS and 1% antibiotics (100 IU penicillin and 100 ug/mL streptomycin) in a humidified incubator at 37°C supplied with 5% CO_2_. In order to explore the effect of ES on L929 fibroblasts, a customized ES chamber was used to apply electrical field stimulation (EFs). The electrotaxis chamber provided a uniform EF across the cells and was connected to the power supply Topward 3303A (Taiwan, China) via agarose salt bridges, potassium chloride saturated salt solution, and Ag-AgCl electrodes. Intermittent direct current EFs were applied at 10V across the chamber (resulting in a field strength of 100mV/mm) for 2h. After that cells were harvested immediately or cultured for another 8h for subsequent experiments. In some experimental sets, BAPTA-AM was added to the medium at the final concentrations 10 *μ*M, incubating cell 30 min before and during the ES.

### 2.3. Intracellular Ca^2+^ Detection

For the free Ca^2+^ measurements, cells were harvested immediately after ES, washed with PBS for 3 times, incubated with Fluo-3AM (10*μ*M) for 30 min at 37°C in the dark, and analyzed by Flow Cytometry (FACSCalibur, BD, USA) at baseline conditions. The concentration of intracellular free Ca^2+^ was recorded as the Geo Mean fluorescence intensity. Experiments were performed for 3 times.

### 2.4. Cell Proliferation Activity Test

To examine the effect of ES on DNA synthesis, EdU was added to the serum-free RPMI 1640 culture media in concentrations of 10 *μ*M for 2h after ES. After the incubation, cells were washed two to three times with PBS, the washes were omitted, and cells were followed by fixation and permeabilization with 4% formaldehyde for 15 min and 0.5% Triton ×100 for 10 min. After washing with PBS, the nuclei were labeled with Hoechst33342. Incorporated EdU was detected by fluorescent-azide coupling reaction. Fluorescence microscope (BX63, Olympus Corp., Tokyo, Japan) was used to detect the fluorescent signals. Experiments were performed for 3 times.

### 2.5. Cell Cycle Analysis

Cells were trypsinized and washed with PBS containing no Mg^2+^ and Ca^2+^ for 3 times and then fixed with precooled 70% alcohol at 4°C overnight. At the second day, cells were washed with PBS and then resuspended in 200 *μ*l, 0.5 *μ*g/ml RNaseA in 37°C for 40 min and then incubated in the darkness at room temperature with 50*μ*l, 50 *μ*g/ml staining solution propidium iodide (PI, Sigma, Louis, MO) for 20 min. The cells were analyzed using the FACSCalibur flow cytometer (BD Biosciences, Franklin Lakes, NJ, USA). Experiments were performed for 3 times. The results were analyzed by Flowjo7.6.

### 2.6. Cell Migration Assays

Cell migration was examined by a Transwell migration assay. Cells were harvested with trypsin immediately after ES and suspended at 3×10^5^ cells/ml. For each group, 200 *μ*l cell suspension was seeded into the upper chambers. The lower contained 10% FBS. After 36h of incubation, some cells on the upper side of the membrane were removed to the lower compartment, followed by washing with PBS for 3 times. The migrated cells on the bottom side of the membrane were then fixed with 4% formaldehyde for 15 min and stained with 0.5% crystal violet (Sigma Aldrich, St. Louis, MO, USA) in 25% methanol for 5 min at room temperature. Six random fields were selected at each image and captured under inverted microscope at 200× magnification and cell number was counted in each field. Experiments were performed 3 times.

### 2.7. Western Blotting

Total protein was extracted from cells using RIPA buffer containing PMSF and phosphorylated protease inhibitors and cocktail (Servicebio, Wuhan, China). Protein was denatured at 95°C for 8 min; then 30 *μ*g of the total protein was analyzed by SDS-PAGE and then transferred onto PVDF membrane. Protein expression levels were incubated with different primary antibodies overnight at 4°C. After that, PVDF membrane was washed with TBST for 3 times and then incubated with Horseradish Peroxidase conjugated IR Dye 800CW and goat anti-rabbit and goat anti-mouse secondary antibodies at 37°C for 1h. The Odyssey Imaging system (LI COR Biosciences, Ltd.) was used for quantification of proteins. Experiments were performed 3 times.

### 2.8. Immunocytochemistry

Cells were washed 3 times with PBS and fixed with 4% paraformaldehyde for 15 min. Coverslips were then rinsed with 0.5% Triton 100× in PBS for 10 min after washing with PBS. After being blocked with goat serum (Servicebio Technology) for 30 min at room temperature, cells were incubated with primary antibody overnight at 4°C. Alexa Fluor 488-conjugated secondary antibodies were used for visualization. The nuclei were labeled with DAPI for 5 min. Images were acquired with a laser scanning confocal microscopy (FV1200, Olympus Corp., Tokyo, Japan).

### 2.9. Mice and Animal Experiment

Virgin female C57BL/6 mice were obtained from the Center of Animal Experiment of Wuhan University. Sixteen virgin female C57BL/6 mice (8~10-week-old) were randomly divided into 2 groups: control and ES group. The mice of ES group were conducted with transvaginal electric stimulation (TES) as we have previously done [13]; the ES parameter was 50HZ, 0.5h×7 days, once per day. All mice were sacrificed and their anterior vaginal tissues were harvested for fixation with 4% paraformaldehyde for 24 h and then embedded in paraffin wax for further study.

### 2.10. Immunofluorescence

All specimens were embedded in paraffin and cut into 4*μ*m thick slices and fixed to glass slides. For immunofluorescence staining, sections were deparaffinized in xylene and rehydrated in a graded ethanol series. Then they were washed with PBS for 5 min×3, along with microwave repair for 8~10 min, and permeated for 10 min in 5% Triton 100×, and then hydrogen peroxide was applied for 15 min, PBS washing along with each step; then goat serum was added to each section for 30 min. After that, the sections were incubated with primary anti *α*-SMA, PCNA, and Vimentin antibodies at 4°C overnight and then with the secondary antibody for 30 min at 37°C. The nuclei were stained with DAPI. Fluorescence microscope (BX63, Olympus Corp., Tokyo, Japan) was used to detect the fluorescent signals.

### 2.11. Statistical Analyses

Statistical analyses were performed with SPSS 22.0 software (IBM Corporation, Armonk, NY, USA), and values were presented as mean ± SD. Differences between two groups were determined using Student's t test, and multiple means were compared by Tukey's test. P values < 0.05 were considered to indicate a statistically significant difference between values.

## 3. Results

### 3.1. ES Increases Intracellular Free Ca^2+^ Level

**Figure 1:**
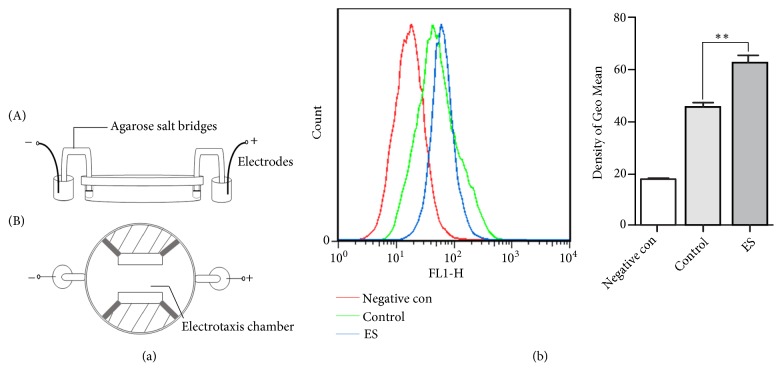
*The effect of ES on intracellular calcium concentration in L929 cell*. (a) Lateral (A) and vertical (B) views of the cell ES device. (b) L929 cells loaded with Fluo-3AM and observed under Flow Cytometry immediately after the cells were treated with ES to evaluate intracellular Ca^2+^ levels (blue); the cells without any treatment were used as the control (green). ^*∗∗*^ P<0.01 compared with the control group. Every experiment was repeated for 3 times. Negative con: negative control group; Control: control group; ES: electrical stimulation group.

Intracellular Ca^2+^ dynamics was detected for the purpose to better understand the response of fibroblast and possible mechanism under ES. ES on the cells was carried out through devices made by us as shown in Figure 1(a). Obviously, in comparison to the control group, the increase in intracellular Ca^2+^ concentration was quite significant higher when the ES was applied as shown in Figure 1(b).

### 3.2. ES Promotes the Proliferation and Migration of L929 Fibroblast

**Figure 2:**
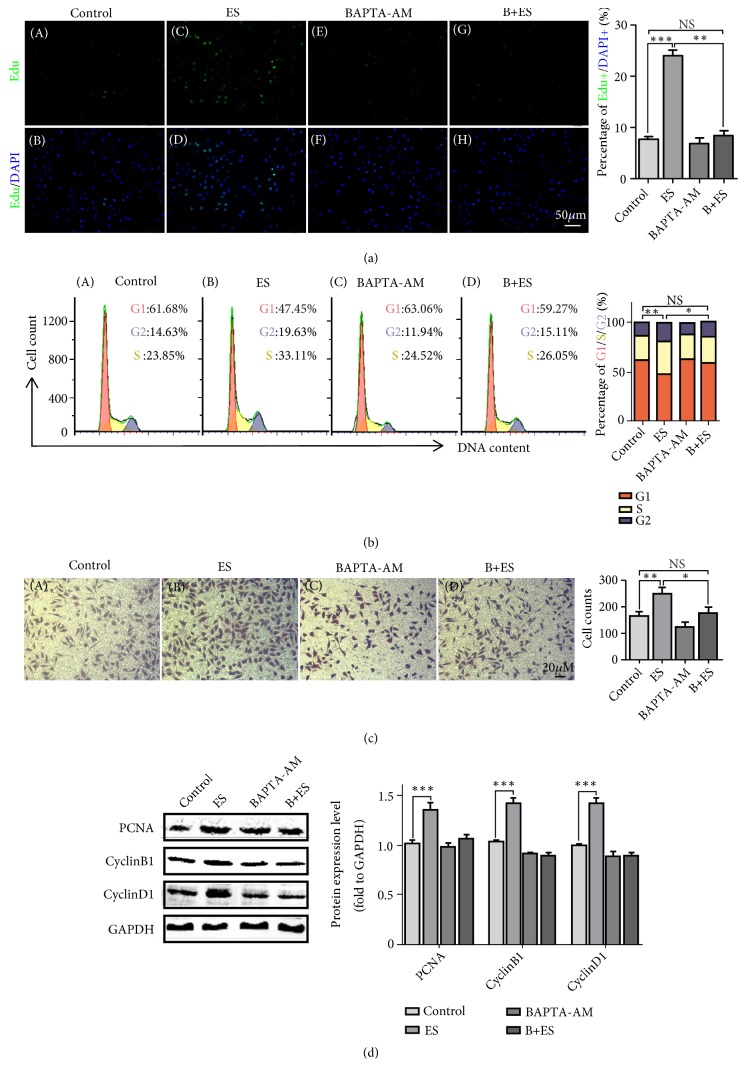
*The effect of ES and BAPTA-AM on cell proliferation and migration*. (a)EdU incorporation was carried on immediately for 2h after ES. Analysis of (b) G1/S/G2 distributions of cell cycle, (c) cell migration, and (d) the expression of proliferation and cell cycle related proteins 24h later after ES treatment. ^*∗∗∗*^ P<0.001; NS: no significance compared with the control group. Every experiment was repeated for 3 times. Control: control group; ES: electrical stimulation group. BAPTA-AM: BAPTA-AM treatment group. B+ES: BAPTA-AM and electrical stimulation treatment group.

Proliferation and migration tendency are important characteristics of fibroblast activation. Therefore, EdU assay and Transwell chamber tests were used to determine the effect of ES on proliferation and migration of fibroblasts. As shown in Figure 2(a) (A–D), the proportion of EdU positive cells significantly increased after ES compared with control. To further study the effects of ES, cell cycle analysis was proceeded on 24 h after ES. As shown in Figure 2(b) (A-B), the percentage of cells in S and G2 phages significantly increased after ES treatment (S phage with19.63% in ES group vs. 14.63% in control; G2 phage with 33.11% in ES group vs. 23.85% in control). In addition, ES promoted the migration of fibroblasts, as shown in Figure 2(c) (A-B); cells passing through the chamber in ES group were significantly increased compared with control group. The PCNA, CyclinB1, and CyclinD1 protein expression levels increased after ES treatment (Figure 2(d)). These results showed that ES can regulate the expression levels of cell cycle proteins and proliferation-related molecules to promote fibroblast cell proliferation activity.

### 3.3. Ca^2+^ Involves in ES Induced Fibroblast Proliferation and Migration

For better understanding of the effect of elevated intracellular Ca^2+^ level induced by ES, BAPTA-AM was used to chelate intracellular free Ca^2+^ in proliferation and migration analysis. As shown in Figure 2(a) (D–G), addition of BAPTA-AM before ES can partially inhibit its effect on cell proliferation promotion. Similarly, the percentage of cells in S and G2 phase (Figure 2(b) (C and D)) and cells passing through the chamber (Figure 2(c) (C and D)) decreased after BAPTA-AM pretreatment followed by ES compared with only ES group. The PCNA, CyclinB1, and CyclinD1 protein expressions were higher with ES treatment and reduced by BAPTA-AM pretreatment (Figure 2(d)). These results reveal that Ca^2+^ involves in ES-induced fibroblast proliferation and migration.

### 3.4. ES Promotes Smad2/3 Signaling Activation, *α*-SMA Expression, and Collagen Deposition of Fibroblasts, and Ca^2+^ May Participate in These Processes

**Figure 3:**
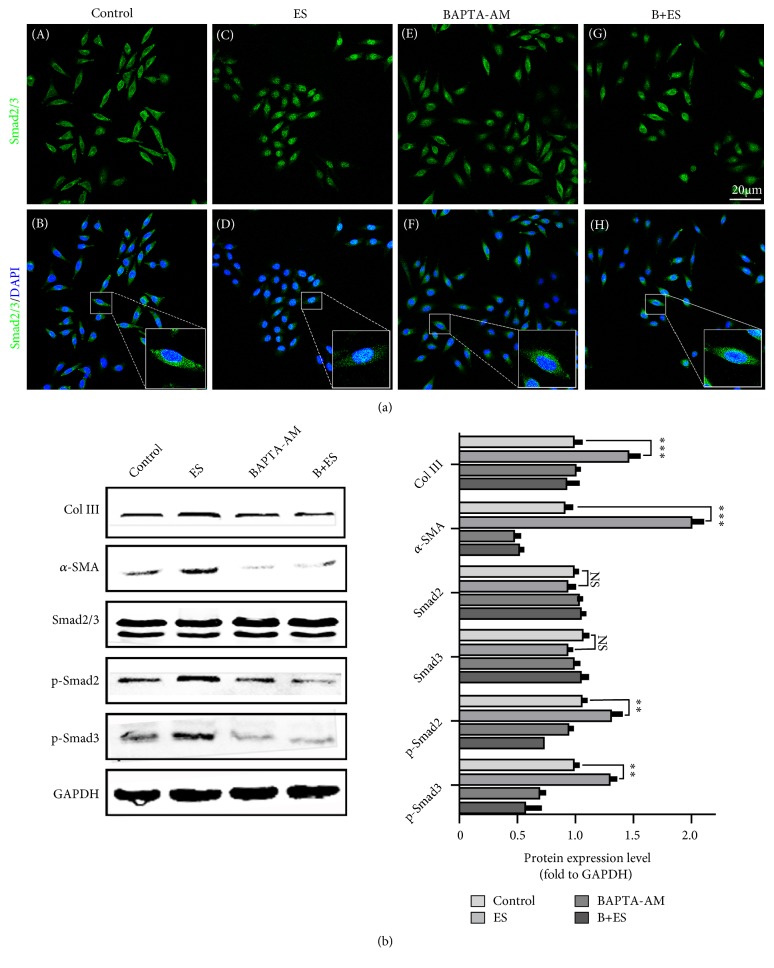
*The effects of ES on Smads signaling and fibroblasts transformation*. (a) The effect of smad2/3 translocation after ES and BAPTA-AM treatment. (b) Col III, *α*-SMA, and Smads signaling related protein expression after ES and BAPTA-AM treatment. ^*∗∗∗*^ P<0.001, NS: no significance compared with control. Every experiment was repeated for 3 times. Control: control group; ES: electrical stimulation group. BAPTA-AM: BAPTA-AM treatment group. B+ES: BAPTA-AM and electrical stimulation treatment group.

Activation of fibroblasts is associated with *α*-SMA expression. Our previous studies have shown that ES promoted the repair of damaged vaginal wall, with enhanced function and increased collagen deposition in anterior vaginal wall, and Smads signaling may be involved in this process [13]. *α*-SMA is an indicator of myofibroblasts transformed from *α*-SMA negative fibroblast. Myofibroblasts have stronger migration and protein secretion ability. In order to further study the function of Ca^2+^ in ES therapy, ES was conducted on L929 cells with or without BAPTA-AM pretreatment; then the effects of ES on Smads signaling and collagen III (Col III) and *α*-SAM protein expression levels were detected. As indicated in Figure 3(a), ES significantly promoted Smad2/3 nuclear translocation, and BAPTA-AM inhibited this process. Besides, ES significantly increased the protein levels of Col III, *α*-SMA, smad2/3, p-smad2, and p-smad3 but is suppressed by BAPTA-AM pretreatment (Figure 3(b)). Obviously, ES elevates Ca^2+^ influx which may play an important role in fibroblast transformation and collagen deposition.

### 3.5. ES Promotes the Activation of Akt/ GSK3*β* Signaling through Elevating Intracellular Free Ca^2+^ Level

**Figure 4:**
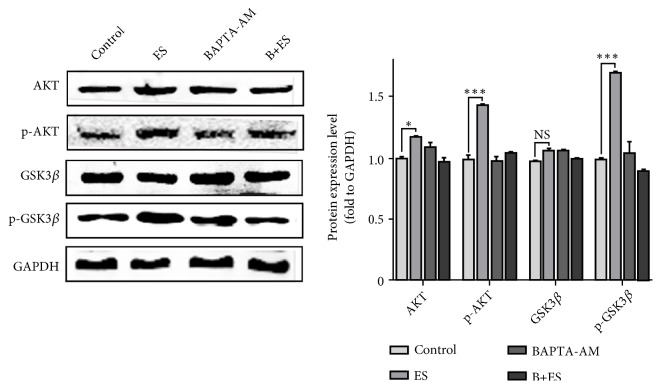
*The effect of ES and Ca* ^*2+*^ *on AKT/GSK3β signaling activation*. The protein expression of AKT, GSK3*β*, p-AKT, and p-GSK3*β* was detected after ES and BAPTA-AM treatment. ^*∗*^P<0.05 and ^*∗∗∗*^P<0.001 compared with control. Every experiment was repeated for 3 times. Control: control group; ES: electrical stimulation group. BAPTA-AM: BAPTA-AM treatment group. B+ES: BAPTA-AM and electrical stimulation treatment group.

Akt signaling participates in many physiological processes, such as cell cycle transition, proliferation, and skeleton rearrangement. In our experiment, the protein level of Akt and its downstream effector, GSK3*β*, were detected. As shown in Figure 4, ES promoted Akt/ GSK3*β* signaling activation via upregulation of Akt and GSK3*β* phosphorylation. In addition, BAPTA-AM pretreatment compromised ES induced Akt and GSK3*β* activation (Figure 4). These experiments confirmed the important role of Ca^2+^ in Akt/ GSK3*β* signaling activation after ES treatment.

### 3.6. ES Promotes the Proliferation and Activation of Fibroblasts of Mouse Anterior Vaginal Walls

**Figure 5:**
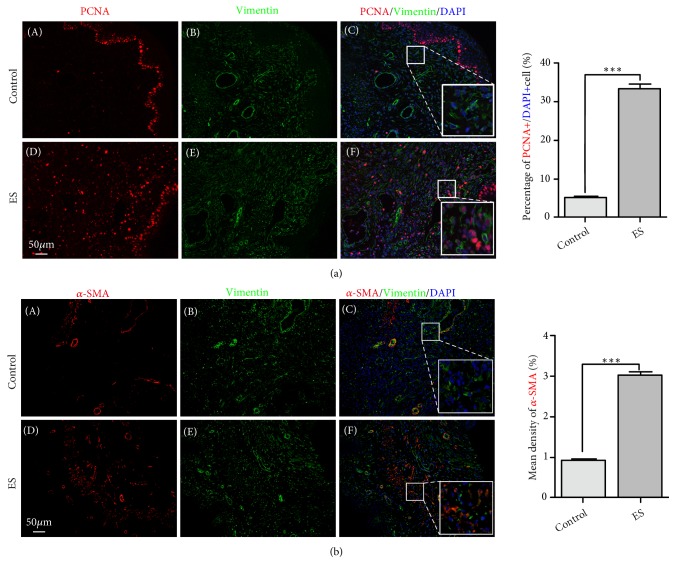
*The effect of ES on mouse anterior vaginal wall*. The effect of ES on PCNA (red) expression (a). *α*-SMA (red) expression (b), with vimentin (green) as an indicator of nonepithelial tissue. ^*∗∗∗*^ P<0.001 compared with the control group; eight mice for each group. Control: control group; ES: electrical stimulation group.

We further studied ES effect on fibroblast* in vivo*. TES mouse model mentioned in our previous study was used in this study. After 7 days, regarding TES, the anterior vaginal wall tissues were collected after the mice sacrifice and protein level of PCNA and *α*-SMA were detected. As shown in Figures 5(a) and 5(b), both PCNA and *α*-SMA protein levels were significantly increased by TES. Anterior vaginal wall of mice from control group showed low protein level of *α*-SMA (Figure 5(b) (A–C)), and ES activated fibroblasts with increased percentage of *α*-SMA positive fibroblasts (Figure 5(b) (D and F)). The* in vivo* experiments mentioned above confirmed that ES can promote the proliferation and activation of fibroblasts, which was the possible mechanism of ES treatment for UI.

## 4. Discussion

The weakness of anterior vaginal wall is one of the manifestations of weak pelvic support and a key factor leading to the occurrence of PFD. Connective tissue and smooth muscle are important composite of the vaginal wall. The abnormalities of the anterior vaginal in UI patients are characterized by the decrease of the collagen component and *α*-SMA [17]. Smooth muscle is the most important component of vaginal wall, playing an irreplaceable role in the function of pelvic support. However, the in-depth study about smooth muscle is lacking since smooth muscle layer of the vaginal wall is deep and the smooth muscle bundles in the pelvic floor ligaments are decentralized. Fibroblast is an important cellular component of ligaments and fascia, which plays an important role in the support, tissue repair, and remodeling after damage. Our previous results confirmed the presence of abnormal collagen metabolism in pelvic ligaments from POP patients [5]. In disease states, decreased quantities of ECM proteins in support tissue lead to reduced resistance to pressures from abdomen and pelvic cavity, contributing to the development of PFD. PES therapy is one of the efficient physical therapies for UI but the mechanism is unclear [18]. Studies have shown that ES could activate fibroblasts and promotes their differentiation and enhances their migration to the site of injury to repair damaged tissue [19–21]. As the effects of ES on cell proliferation, orientation, and differentiation, it has been widely used in the field of genetic engineering to regulate tissue regeneration [22].

Activation of fibroblast and increased collagen deposition should be important for the repair of damaged vaginal wall. To elucidate the mechanism of ES in the treatment of SUI and clarify the effects of ES on fibroblasts, L929 cells were used in our* in vitro* experiments. ES on cells is usually conducted with electrotaxis chambers and the parameters are within the range of 50 to 100 mV/mm [23, 24]. We use similar devices, applying 50mV/mm, 100mV/mm, and 200mV/mm for 2 to 6 hours, and finally obtain the most appropriately stimulus parameters without any damage effect being 100mV/mm for 2 hours (data not shown). Although fibroblasts are nonexcitable cells, early studies have found that they have a voltage-dependent calcium channel on the cell surface [11], so we measured the intracellular Ca^2+^ level with and without ES. Obviously, ES significantly elevates the level of intracellular Ca^2+^ as shown in Figure 1. The elevation of cytoplasmic Ca^2+^ could be through extracellular Ca^2+^ flow upon the opening of voltage-dependent calcium channel, intracellular Ca^2+^ regulation pathways, store-operated Ca^2+^ entry, or the transient receptor potential cation channels, TRPC on the surface of cell membranes [25–27]. But we did not study the detailed modulation mechanisms of them in this article.

In normal conditions, the basal concentration of intracellular Ca^2+^ is maintained at very low levels compared to the extracellular spaces. As an important second messenger, the change of cytoplasmic Ca^2+^ is involved in various physiological and pathological processes [28]. The elevated cytoplasmic Ca^2+^ level could regulate the cytoskeleton rearrangement, as well as the activation and differentiation of fibroblasts. Wei Zhang et al. demonstrated that inhibition of calcium-calmodulin-dependent protein kinase II (CaMKII), one of the main protein kinases mediating intracellular Ca^2+^ changes, suppressed cardiac fibroblast proliferation and Col I/III secretion induced by ES [29]. Chelating external Ca^2+^ by EGTA prevents the proliferation and enhances superoxide production of cultured rat cardiac fibroblasts [30]. In addition, mibefradil, a mixed calcium channel blocker, compromised collagen production and differentiation ability of fibroblast in rats [31]. During fibroblast cell migration, the distribution of Ca^2+^ changes with directional movement [31]. These studies suggest that Ca^2+^ is important for fibroblast responses and tissue remodeling and may be an indispensable regulator in ES treatment.

Furthermore, after ES treatment, altered calcium signaling is associated with the improvement of proliferation, differentiation, and migration. Smads signaling is important in ECM metabolism in fibroblasts and may be involved in PES treatment [13]. So, we use BAPTA-AM, a permeable free Ca^2+^ chelator, to prevent free Ca^2+^ mediated effect and verify the crucial role of Ca^2+^ in ES induced smad2/3 nuclear translocation. Similar to our conclusion, Wei L et al. showed that Ca^2+^ played a role in Smads signaling activation in cardiac fibrosis [32]. Akt pathways were related to the proliferation and differentiation of fibroblasts and associated with cell cycle progression and migration [15, 16]. Therefore, we simultaneously tested its expression and activation in our study. The results showed that Akt/GSK3*β* signaling was active by ES and its activation was relevant with the elevation of cytoplasmic Ca^2+^ levels. The activation of Smads and Akt/GSK3*β* signaling may be mediated by Ca^2+^ in cytoplasm and in turn activates a series of downstream effectors. In addition, studies have shown that Ca^2+^ distribution is regulated by a variety of signaling molecules such as TGF-*β* [33]. How does Ca^2+^ regulate the switch of signaling pathway and whether there is a feedback regulation mechanism? Further study is needed.

Mice study was also used in this study to verify the effects of ES on fibroblast proliferation and activation* in vivo*. PCNA is a protein participating in cell cycle regulation and is involved in ongoing DNA replication. *α*-SMA is recognized as an indicator for fibroblast phenotypic transformation. In our results, transvaginal PES promoted the expression of PCNA and *α*-SMA in anterior vaginal wall of mice. Their upregulation suggested the proliferation and activation of the fibroblasts after ES, which may be the mechanism of PES therapy.

## 5. Conclusion

To sum up, in this study, we verify that ES can elevate the concentration of intracellular Ca^2+^ and then promote the activation of fibroblasts. Besides, Smads and Akt/GSK3*β* signaling may be involved in Ca^2+^ mediated activation of fibroblasts after ES. The increase of intracellular Ca^2+^ deriving from extracellular Ca^2+^ flow from the opening of calcium channels on the membrane or intracellular calcium store release upon ES needs further exploring.

## Data Availability

The data used to support the findings of this study are included within the article.
